# A Digital Tool for Culturally Responsive Reflection and Case Conceptualization in First-Line Services to Support Children With a Refugee Background: Development and Usability Study

**DOI:** 10.2196/86809

**Published:** 2026-10-15

**Authors:** Charlotte Angela Van Bogaert, Benji Jame, Geertrui Maria Serneels, Tom Van Daele

**Affiliations:** 1 Psychology and technology, Centre of Expertise Care and Well-being, Thomas More University of Applied Sciences, Molenstraat 8, Antwerp, 2018, Belgium, 32 3 432 18 62; 2 Solentra vzw - University Hospital Brussels, Brussels, Belgium; 3 TIME Centre for Technological Innovation, Mental Health and Education, Queen’s University Belfast, Belfast, United Kingdom

**Keywords:** refugees, child mental health, cultural competence, digital decision support, qualitative research

## Abstract

**Background:**

In 2023, over 117 million people were forcibly displaced worldwide, 40% of them children. Refugee children are at higher risk for posttraumatic stress disorder, depression, and anxiety, while professionals may struggle to address mental health and trauma-related concerns across unfamiliar cultural contexts. The Psychiatry Assisting the Culturally Diverse Community in Creating Healing Ties (PACCT) approach supports culturally responsive care for culturally diverse families, but first-line professionals often lack practical support to apply such an approach in routine care.

**Objective:**

The study aimed to (1) identify professionals’ support to inform the development of Implementing PACCT through a Culturally Contextualized Tool (IMPACCT), a digital decision-support tool; (2) map these needs onto the initial IMPACCT conceptualization; and (3) gather initial feedback from professionals to guide further refinement.

**Methods:**

We conducted a qualitative descriptive study in 3 phases. First, we analyzed 905 anonymized help desk questions submitted between May 2021 and November 2023 to Solentra, a Belgian center of expertise providing transcultural mental health support and consultation for refugees and other vulnerable migrant populations. Questions were coded using a predeveloped framework to identify recurrent professional needs. Second, we mapped all help desk question categories onto the different components of the first conceptualization of IMPACCT to ensure that its content reflected users’ questions and expectations. Third, 19 professionals working with refugee and migrant populations in Belgium were recruited through an open call between December 2024 and February 2025 using convenience sampling. After hands-on experience with a first iteration of IMPACCT, they provided feedback during semistructured interviews. These interviews were subsequently analyzed using deductive qualitative content analysis.

**Results:**

In the help desk questions, trauma-related concerns were the most common, followed by anxiety/stress and depressive symptoms. Acculturation was the most dominant culture-migration category. Most contexts were family- and school-oriented, and professionals were primarily asking for supervision or concrete next-step guidance. The interviews (N=19) found that the professionals thought that IMPACCT provided them with better-structured and early reflection, lessened some of the decision-making burden, and enabled a more structurally informed understanding of culture in the context of migration. Reported barriers included the time required and a lack of adaptation in workflow, with more experienced professionals reporting less perceived value. Additionally, participants suggested using IMPACCT as a training and/or supervision aid in first-line services and as standard decision support in ambulatory and inpatient care.

**Conclusions:**

The study offers first insights into the possible conceptualization of a digital decision-support tool for first-line professionals to help them support children with a refugee background. Further technical optimization of the tool, also taking into account first-user feedback, is, however, necessary before subsequently exploring its efficacy as well as effectiveness in strengthening first-line professionals’ competencies in working with the vulnerable population.

## Introduction

### Background

At the end of 2023, a record 117.3 million people worldwide were forcibly displaced due to persecution, conflict, violence, human rights violations, or events seriously disturbing public order, representing an 8% increase from 2022 and 12 consecutive years of increasing forced displacement. Children represent 40% of all forcibly displaced people, while making up only 30% of the world’s population [1]. Previous studies have characterized children with a refugee background as a vulnerable minority group that has an increased risk for developmental trauma as a result of violence or instability in their country of origin and hazardous journeys to the host country [2]. Developmental trauma refers to the effects of chronic early-life adversities such as prolonged exposure to violence or displacement that disrupts a child’s emotional, cognitive, and social development. Children with a refugee background have, therefore, unsurprisingly been found to be at an elevated risk of posttraumatic stress, depression, anxiety, and difficulties with school functioning, particularly in the absence of coordinated, culturally responsive support [3]. In addition to exposure to potentially traumatic events, children face ongoing migration- and acculturation-related stressors. Forced migration often comes with experiences of loss, uprootedness, and disconnection. Although mental health problems may arise from profound psychological injury such as trauma, they can just as well be a normal and adaptive response to an abnormal context of forced displacement and acculturation.

Professionals who work with children with a refugee background (eg, teachers, youth care workers, and clinicians) frequently experience difficulties engaging with mental health problems and trauma-related behaviors in cultural contexts with which they might not be familiar [4]. Studies suggest they frequently report feeling unprepared to deal with mental health issues in refugees and asylum seekers due to factors such as cultural and language barriers [5-7]. While some training exists, it is often too general or fails to provide actionable strategies tailored to culturally diverse, trauma-affected populations. Moreover, there is a notable scarcity of validated tools to support these professionals in working with refugee children less than the age of 6 years [8]. This makes it harder for professionals to distinguish between profound psychological injury and normal stress reactions and to determine appropriate next steps [9].

As a first step in response to the need for culturally competent and sensitive mental health care, the Psychiatry Assisting the Culturally Diverse Community in Creating Healing Ties approach (PACCT) was created as a community-based intervention model to work with culturally diverse families [10]. PACCT emphasizes that effective transcultural, culturally sensitive, and trauma-sensitive care always starts from and engages with the child’s community. The framework provides some guidance for professionals by delivering context-sensitive engagement with refugee families. It intends to create a mutual understanding of the child’s behavior and distress (Multimedia Appendix 1). However, despite some potential clinical success when using the PACCT methodology, a gap remains between the promise of PACCT and its implementation, as it often relies on in-person guidance from specialized teams, which limits its uptake in broader primary and secondary care contexts.

The past 2 decades have seen growing interest in the use of digital mental health interventions for a wide variety of purposes and populations [11]. In recent years, this interest has also extended to refugee populations, with most interventions being patient-facing and focusing on psychoeducation, self-management, symptom monitoring, and treatment delivery [12,13]. At the same time, digital decision-support systems have increasingly been explored in broader mental health settings to assist professionals with assessment, triage, risk identification, and treatment planning. Such systems may help structure clinical reasoning, reduce uncertainty, and support more consistent decision-making [14,15]. However, the application of such approaches to support frontline professionals working with refugee children and families remains largely unexplored [12,13]. Consequently, there is limited evidence regarding how digital decision-support tools can help professionals interpret mental health concerns and determine culturally and trauma-sensitive responses in this population.

### Objectives

The aims of this study are, therefore, (1) to identify current support needs of professionals working with refugees in order to inform the development of IMPACCT, a digital decision-support tool; (2) to map needs to the conceptualization of IMPACCT; and (3) to gather initial feedback from professionals to inform further development and refinement of IMPACCT for broader use in practice.

## Methods

### Study Design

This qualitative descriptive study relied on three data sources: (1) an analysis of existing help desk questions; (2) a mapping of identified categories onto the different steps of the IMPACCT; and (3) semistructured interviews with professionals who piloted a first conceptualization of the IMPACCT. The SRQR (Standards for Reporting Qualitative Research) reporting guideline [16] was used to guide the reporting (Checklist 1).

### Phase 1: Help Desk Questions

#### Data Source and Sample

Help desk questions were submitted to Solentra through an online form by professionals seeking advice on clients with a refugee background whom they were currently supporting or treating. The questions consisted of free-text descriptions written by the professionals themselves and concerned concrete cases from ongoing practice. The dataset covered clients across age groups, including both children and adults. All 930 questions were collected between May 2021 and November 2023 and anonymized by Solentra before being provided to the research team for analysis. After initial cleaning, 25 questions were excluded due to insufficient information (n=2), duplication (n=17), submission in a language other than Dutch, English, or French (n=4), or both duplication and language incompatibility (n=2). In total, 905 unique questions were retained for analysis.

#### Data Analysis

Help desk questions were analyzed according to a fixed coding structure, developed by an author with extensive professional experience in refugee mental health (BJ), drawing on prior work and domain expertise. A full overview of the coding structure, including all subcodes, can be found in Table 1.

**Table 1.** Analysis of help desk questions (N=905)^a^.

Coding category	Frequency, n (%)	
Adverse experience	520 (57.5)	
Clinical concerns	577 (63.8)	
Trauma symptoms	282 (31.2)	
Anxiety and/or stress symptoms	130 (14.4)	
Behavioral problems	130 (14.4)	
Depressive symptoms	91 (10.1)	
Learning problems	58 (6.4)	
Sleep problems	43 (4.8)	
Psychosis	42 (4.6)	
Suicidality	35 (3.9)	
Psychosomatic symptoms	31 (3.4)	
Autism (spectrum disorder)	30 (3.3)	
Substance use	20 (2.2)	
Self-harm	19 (2.1)	
Attachment problems	17 (1.9)	
Physical disability	9 (1.0)	
Culture and migration	304 (33.6)	
Acculturation process	235 (26.0)	
Grief	47 (5.2)	
Cultural interpretation	38 (4.2)	
Radicalization	6 (0.7)	
Systemic/social context	438 (48.4)	
Family problems	253 (28.0)	
School or work problems	160 (17.7)	
Legal difficulties	48 (5.3)	
Social isolation	44 (4.9)	
Social contacts	36 (4.0)	
Care pathway	548 (60.6)	
Referral	304 (33.6)	
Next steps	294 (32.5)	
Diagnostics	30 (3.3)	
Professional needs	852 (94.1)	
Supervision/support	842 (93.0)	
Training	7 (0.8)	
Feelings of hopelessness	3 (0.3)	

a Percentages were calculated by dividing the frequency of each subcategory by the total number of valid cases (N=905). Because multilabel coding was applied, subcode totals can exceed their parent category totals.

Within each category, raters could select any relevant subcode. Most subcodes were self-explanatory, but for some, additional information was provided. For each of the 905 help desk queries, raters were first asked to mark the first category, *adverse experience,* as either present or absent. This was defined as exposure to a harmful event (eg, violence, bereavement, forced migration, or bullying). Forced displacement in itself was seen as an adverse experience. Subsequently, each query was rated across 5 additional categories. The second category was *clinical concerns,* which consisted of 14 different subcategories. For the second categories, trauma symptoms and suicidality, additional instructions were provided. Trauma symptoms were only coded when the query explicitly identified the experience as trauma or clearly described trauma symptoms. In line with the *Diagnostic and Statistical Manual of Mental Disorders, 5th Edition* (*DSM-5*), we primarily coded trauma when intrusive symptoms or avoidance (criteria B and C) were described. Changes in cognitions and mood or arousal and reactivity (criteria D and E) are often more difficult to directly link to trauma, as they may also occur within anxiety or depression. These were therefore only coded as trauma symptoms when B or C were also present, or when the experience was explicitly linked to a psychotraumatic event [17]. Suicidality was coded when there was any mention of suicidal thoughts and/or behavior or suicide attempts, past or present. The third category, *culture and migration,* had additional instructions for acculturation, cultural interpretation, and grief. Acculturation was defined as the cultural and psychological adaptation that occurs when individuals arrive in a cultural system that is different from their original one. It involves learning and using the host culture’s language, norms, and practices while retaining some parts of the origin culture (to varying degrees) [18]. It was coded when there were any direct or indirect mentions of an adjustment process related to building a new life in the new country. Cultural interpretation was coded when professionals posed questions on the cultural interpretation of symptoms, problems, and solutions. Grief was coded when there was any mention of difficulties related to experiences of loss. The remaining categories were *systemic and social context*, *care pathway,* and *professionals’ needs*.

To assess interrater reliability, three raters (CVB, BJ, and TVD) independently coded 70 help desk questions. This consisted of an initial calibration set of 50 questions and an additional set of 20 questions following discussion of discrepancies and clarification of coding definitions. Fleiss κ was calculated for each overarching category. The reliability assessment was used as a calibration procedure to identify categories requiring further clarification, rather than as a formal stopping rule. After the calibration phase, BJ coded the next 250 questions, and CVB coded the remaining ones.

### Phase 2: Mapping of Help Desk Questions

#### IMPACCT Development

Based on the PACCT methodology, a first conceptualization of the IMPACCT was developed as an educational and reflective tool for first-line professionals working with refugee children and families. It was not intended to provide diagnoses or compulsory advice, but rather to support case reflection, strengthen professionals’ understanding of relevant psychosocial and cultural factors, and help them consider when additional support or referral may be warranted. Its key components included an initial cultural sensitivity check, followed by questions introducing an ecological perspective. Subsequent sections addressed acculturation, grief, trauma, and resilience. The tool concluded with information on potential referral options and final guidance.

#### Data Analysis

All help desk question categories were subsequently mapped onto the different components of the IMPACCT to ensure that its content reflected users’ questions and expectations. Based on this mapping, topics were classified as (1) well represented, (2) partially represented, or (3) underrepresented. Well-represented topics were those that were frequently raised in help desk questions and were directly addressed within the tool. Underrepresented topics, by contrast, concerned content considered important within the PACCT framework but that had been raised relatively infrequently by users. This classification helped to further refine the content of the tool. In particular, for partially represented and underrepresented topics, additional attention was given to explaining their relevance and why professionals should consider them even when they are not explicitly raised by users.

### Phase 3: Semistructured Interviews

#### Participants

All participants were professionals working in health care, welfare, education, or community-support settings in Flanders, Belgium, including, but not limited to, general practitioners, primary care psychologists, school counselors, and social workers. They were recruited through an open call disseminated by Solentra and partner organizations via mailing lists and social media posts in November and December 2024.

This convenience sampling strategy resulted in 38 professionals expressing interest, of whom 19 participated between December 2024 and February 2025. Reasons for nonparticipation following the initial expression of interest could not be determined, as these professionals did not respond to subsequent email contact. The final sample size was considered sufficient for a pragmatic first-user evaluation aimed at collecting feedback across a range of professional practice settings.

#### IMPACCT

The tool that participants assessed was a responsive website, through which they first submitted basic background information. Before proceeding to the case exploration, users were guided through a brief cultural sensitivity reflection. This section asked whether the professional had reflected on their own cultural position, whether the family understood the Western care context, and whether there was a possible need for an interpreter. Each question included an explanatory *“Why is this important?”* pop-up, and the tool offered additional resources on cultural sensitivity and interpretation where relevant.

The next phase of the tool was structured around an ecological perspective following Bronfenbrenner [19]. Rather than focusing exclusively on the child, IMPACCT invited professionals to map concerns, stressors, and resources across the child’s broader context. This ecological mapping was organized across several layers: at the individual level, the microsystem, the mesosystem, the exosystem, and the macrosystem. Explanatory pop-ups again clarified which ecological layer was addressed at each step and explained the rationale for the questions posed (Figure 1).

**Figure 1.**
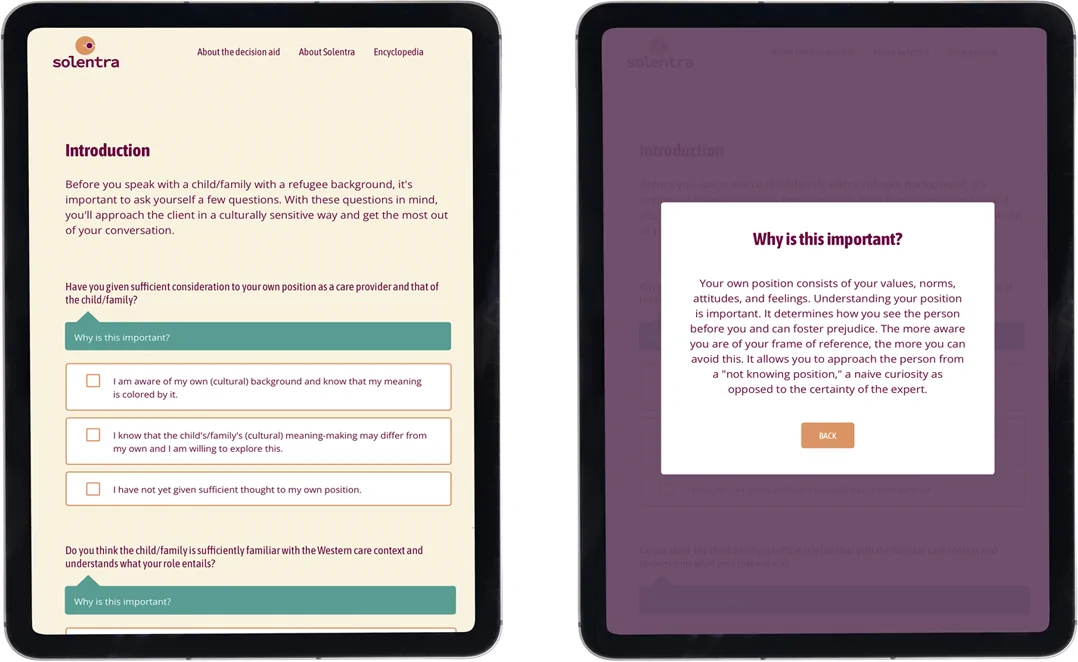
Multiple-choice questions including the option to have key concepts clarified through pop-ups. The language in the screenshots has been translated from Dutch to English for publication purposes.

The tool subsequently explored 4 central themes: acculturation, grief, trauma, and resilience (Figure 2). The tool concluded with personalized feedback (Figure 2) for professionals. IMPACCT also included an encyclopedia with background information on themes such as acculturation, grief, trauma, resilience, and stress reduction. The tool was expected to be completed in approximately 10 to 15 minutes, although this was not evaluated in the present study. A more detailed description of the tool can be found in Multimedia Appendix 2.

**Figure 2.**
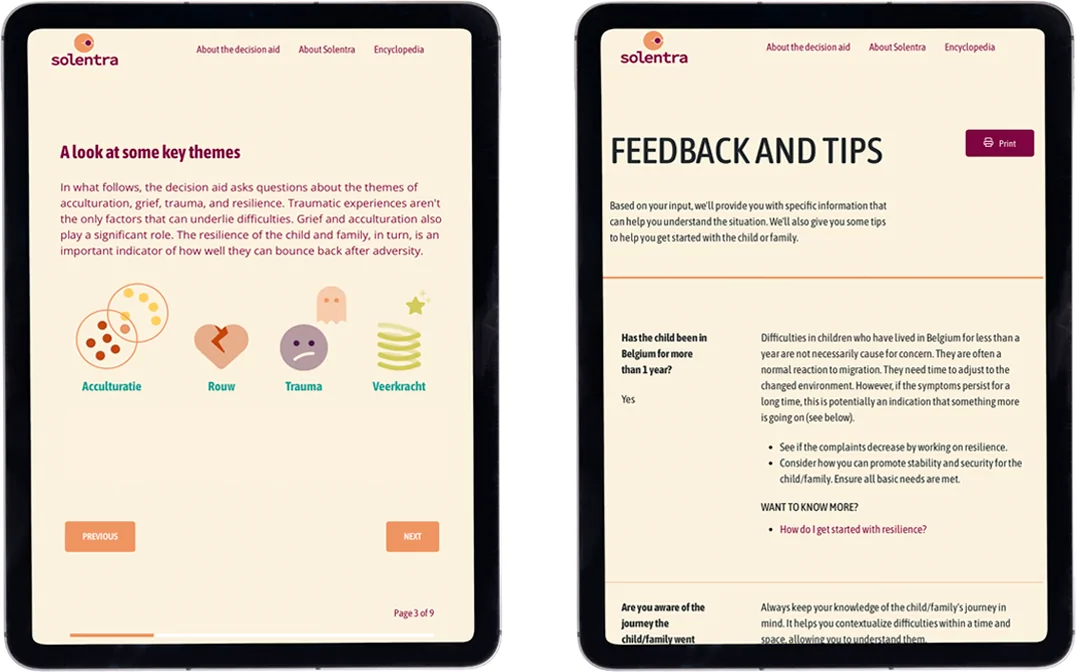
Introduction of key themes and final page of Implementing PACCT through a Culturally Contextualized Tool (IMPACCT), summarizing answers and offering concrete feedback and tips. The language in the screenshots has been translated from Dutch to English for publication purposes. PACCT: Psychiatry Assisting the Culturally Diverse Community in Creating Healing Ties approach.

#### Procedure

Professionals who expressed interest through an online form were contacted by email and provided with access to IMPACCT for a hands-on session. Participants accessed a web-based prototype of IMPACCT remotely through a standard web browser on their personal device. They were invited to explore the tool with a recent case from their professional practice in mind.

Following this hands-on experience, participants took part in a semistructured interview (Multimedia Appendix 3). The interview covered sociodemographic information and participants’ experiences with IMPACCT, including user experience, content and impact, as well as practical relevance. The semistructured interviews were conducted by 6 bachelor-level student researchers under the supervision of the research team. A total of 12 interviews took place online via MS Teams, and 7 were conducted in person at a location of the participant’s preference. With participants’ consent, interviews were recorded either via MS Teams or using audio-recording software and subsequently transcribed verbatim.

#### Data Analysis

Semistructured interviews were transcribed verbatim and analyzed using deductive qualitative content analysis. The coding framework was developed by the research team beforehand and consisted of 5 predefined categories, allowing for subcodes to emerge inductively within each. These categories reflected the aims of the first-user evaluation and the topics addressed in the interview guide. The first category was *usability and workflow fit*, which related to the interface, first impressions, and ease of use. The second category was *content relevance and cultural fit* clustered subcodes on clarity, case relevance, fit with atypical family contexts, and parent/child perspective. The third category was *practical impact and decision support* focused on whether the use of the tool had an impact on professionals’ actions or discussions. The fourth category was *perceived added value* related to whether professionals considered the tool useful and whether or not they would recommend it. Finally, *suggestions for improvement* concerned concrete proposals for what could be changed or improved on the tool. CVB coded all transcripts by assigning relevant text segments to one or more of the predefined categories. The coded material was then reviewed by TVD to assess whether the categorization was consistent with the predefined framework and whether the selected findings adequately reflected the interview data. Uncertainties in categorization or interpretation were discussed between CVB and TVD until agreement was reached.

### Ethical Considerations

The study was approved by the Ethical Committee of the Department of Applied Psychology at Thomas More University of Applied Sciences (ECTP2425_07). Help desk questions were only made available as an anonymous dataset. Interview participants were informed about the objectives, design, and data processing both verbally and through an information letter. All interviewees provided written informed consent before participating. Participation was voluntary, and as a token of appreciation, participants had the option to receive a €25 (€1=US $1.05 as of September 30, 2026) gift certificate. Interview data were pseudonymized during transcription and analysis. Data were stored securely and were accessible only to the research team. Identifying information was removed during transcription, and pseudonymized transcripts were used for subsequent analysis. Audio recordings and transcripts were stored securely and were accessible only to the research team.

## Results

### Phase 1: Help Desk Questions

A total of 905 help desk questions were coded. Across the 70 items independently coded by all 3 raters, there was almost perfect agreement for *adverse experience* (κ=1.00), substantial agreement for 4 categories (0.67<κ<0.78), and moderate agreement for *culture and migration* (κ=0.57). Because culture and migration showed lower agreement than the other categories, findings for this category were interpreted cautiously.

Table 1 offers an overview of the queries. In over half of them, an *adverse experience* was explicitly mentioned. Presentations of *clinical concerns* were frequent and heterogeneous. Trauma-related symptoms were the most common clinical concerns, appearing in one-third of all cases (and in half of the instances where clinical concerns were mentioned). Anxiety and/or stress symptoms were mentioned in just under one in seven, and depressive symptoms in about one in ten. Co-occurrence was common. Many queries could be linked to the topic of *culture and migration*, often referencing the acculturation process. To a lesser extent, there were also questions around grief and cultural interpretation. When *systemic and social context* was coded, the most frequent contexts were family problems, followed by school/work challenges, legal difficulties, social isolation, and difficulties in social contacts. Very often, questions related to the *care pathway*. Most queries sought a referral; a comparable share asked for actionable next-step guidance. A smaller subset requested diagnostic help, often due to concerns that standard Western instruments might be inappropriate or unfair given language barriers or differences in cultural knowledge. Finally, almost all questions were related to one or more *professional needs*. The most common request was for supervision (also the main intended purpose of the help line), referring to case guidance to improve decision-making, quality, and worker well-being across cross-cultural situations. A minimal number of questions also involved requests for training or expressions of feelings of hopelessness.

The analysis of help desk questions revealed recurring patterns highlighting both pressing needs and overlooked areas among professionals. Clinical concerns, particularly trauma symptoms, surfaced repeatedly, confirming the need for a tool that helps professionals consider how migration-related stress, grief, and trauma can shape children’s well-being. Systemic and social context factors, most often linked to family and school life, also appeared frequently, showing that concerns rarely occur in isolation but are embedded in children’s wider environments. Issues around acculturation were raised regularly as well, pointing to the challenges families face in finding their place in the new social network, learning the language, and navigating life between two cultural systems. Care pathway questions typically focused on “what to do next” or on referral, underscoring the need for structured guidance rather than diagnostic tools. Finally, the overwhelming demand for supervision can be seen as a clear need of professionals for support in decision-making, alongside opportunities for building knowledge and confidence in working with children with a refugee background.

In contrast, several categories were less common, such as grief. The high occurrence of trauma symptoms suggests that professionals tend to link a wide range of symptoms to trauma rather than to categories such as grief or acculturation, which are more specific to the refugee experience. Although issues around acculturation were identified regularly in the help desk questions, professionals generally described them as issues related to integration, rarely framing it within an acculturation process or linking this process with symptoms of stress, anxiety, or depression. Finally, help desk questions largely focus on issues and everything that is going wrong but less on signs of resilience.

### Phase 2: Mapping of Help Desk Question Topics

The mapping of help desk question topics onto the different components of the IMPACCT (Figure 3) found that the tool covers nearly all identified topics, with the exception of radicalization. Although the tool addresses underlying mechanisms such as trauma, social isolation, and identity confusion, radicalization itself was considered beyond its intended scope. Of the remaining topics, 11 were classified as well represented, 4 as partially represented, and 6 as underrepresented. Topics that were partially represented or underrepresented received additional attention in the tool, particularly through further clarification of their relevance to professional practice.

**Figure 3.**
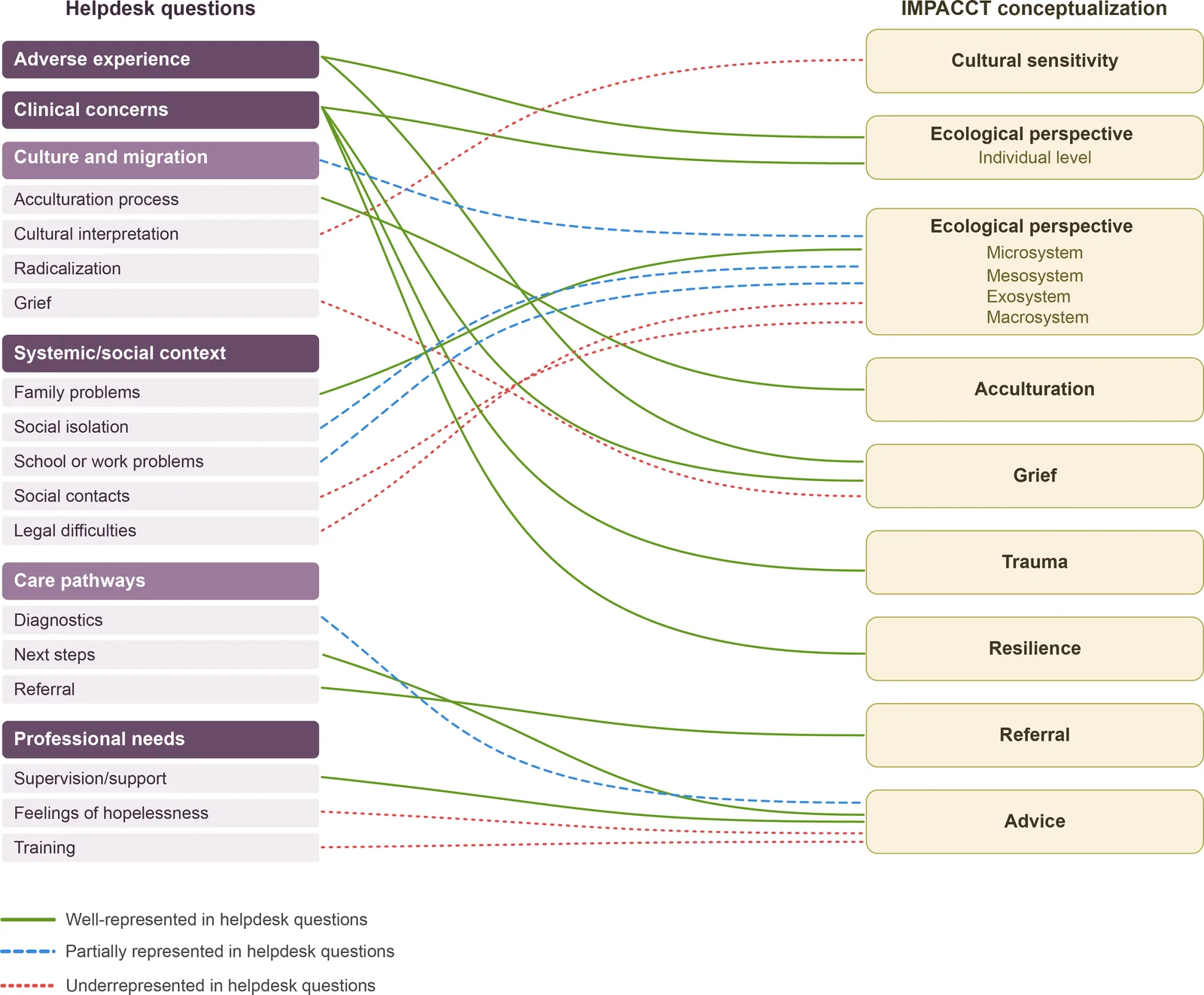
Mapping of categories from Solentra Help desk question topics onto the different components of the Implementing PACCT through a Culturally Contextualized Tool (IMPACCT). PACCT: Psychiatry Assisting the Culturally Diverse Community in Creating Healing Ties approach.

### Phase 3: Semistructured Interviews

#### Participants

Nineteen participants (16 women and 3 men) agreed to a hands-on experience with the tool, followed by an interview. The mean age was 45.5 (SD 8.2; range 31‐58) years, with an average of 16.3 (SD 7.4; range 4‐35) years of professional experience. The participants represented a broad range of practice settings. In the clinical and therapeutic setting, the sample included clinical psychologists and a crisis counselor. Within the social work and community care setting, participants ranged from family support workers to guardians for unaccompanied refugee minors. The policy and coordination group comprised roles such as a head of diversity and inclusion and coordinators of family support centers. Finally, under education and training, 2 lecturers in social disciplines were included. Detailed characteristics are provided in Table 2.

All interviews were analyzed across five categories: (1) usability and workflow fit, (2) content relevance and cultural fit, (3) practical impact and decision support, (4) perceived added value, and (5) suggestions for improvement.

**Table 2.** Participants’ characteristics (N=19).

ID	Age (y)	Gender	Experience (y)	Practice setting
ID1	43	Man	10	Clinical and therapeutic
ID2	53	Woman	13	Policy and coordination
ID3	56	Woman	8	Education and training
ID4	48	Woman	23	Policy and coordination
ID5	49	Man	4	Nursing/psychotherapy
ID6	52	Woman	15	Social work and community care
ID7	58	Woman	20	Social work and community care
ID8	50	Man	20	Policy and coordination
ID9	39	Woman	19	Policy and coordination
ID10	58	Woman	35	Clinical and therapeutic
ID11	34	Woman	9.5	Social work and community care
ID12	43	Woman	15	Social work and community care
ID13	31	Woman	10	Clinical and therapeutic
ID14	44	Woman	20	Social work and community care
ID15	42	Woman	17	Nursing/psychotherapy
ID16	47	Woman	25	Policy and coordination
ID17	35	Woman	12	Education and training
ID18	47	Woman	24	Policy and coordination
ID19	35	Woman	10	Clinical and therapeutic

#### Category 1: Usability and Workflow Fit

Participants consistently described the interface as clear and easy to navigate, with embedded concept explanations being particularly valued: “Yes, it was a very nice website, fancy too. Definitely user-friendly.” (ID1), “The little explanation boxes next to difficult words were helpful.” (ID9). However, time investment was considered the main barrier: “Very easy to use...but you should use the tool when you have enough time.” (ID14), “Takes some time and energy.” (ID11). This suggests a difference in technical usability and practical feasibility. Although participants generally found the tool easy to operate, its length could limit its use in time-pressured settings.

#### Category 2: Content Relevance and Cultural Fit

Participants highlighted value in prompted reflection on one’s own reference frame and in reframing behavior within the migration context. One user explained, “This tool likely helps with self-reflection...it also confronts us with the fact that we all have our own frames of reference...It can broaden anyone’s perspective.” (ID7). Similarly, another user stated that “The tool can be used to raise awareness on refugee background; it can then help you reflect.” (ID11). These responses suggest that the tool’s perceived value lay not only in providing information but also in encouraging professionals to reconsider assumptions that may shape their interpretation of a case. After using the tool, participants described becoming more attentive to migration and flight experiences, social networks both in Belgium and in the country of origin, and resilience alongside trauma and loss (ID 7, 11, 14, and 17).

At the same time, participants identified gaps in the tool’s ability to accommodate more complex or atypical cases. For example, one participant described a case in which the parent did not function as a source of support: “The parent was actually more of a burden to the child in this case than a source of support.” (ID1). Similar difficulties were reported when working with unaccompanied refugee minors: “Involving the parents of unaccompanied refugee minors is not simple.” (ID10), while another participant explained that information about parents, loss experiences, and support figures was “often not entirely clear for this population.” (ID14).

#### Category 3: Practical Impact and Decision Support

The tool facilitated early reflection and clarified thresholds for action/referral; participants described it as a useful filter when they were unsure: “When you’re stuck with a case, it can give some direction on what needs to be done, for example, making a referral.” (ID4), “The recommendations or information that appeared...provided added value.” (ID19). Participants also described potential value in using the tool during collaborative or multidisciplinary case discussions (ID 4, 14, and 16). One participant suggested using it to consider “What are we already doing, who can do what, do we need to refer, and what can we take on ourselves?” (ID14). These responses suggest that the tool could support both individual decision-making and collaborative case discussions.

#### Category 4: Perceived Added Value

A minority reported limited incremental value in highly experienced settings. These views generally co-occurred with calls for tighter workflow fit or richer, case-specific guidance: “Not for the time being, because it doesn’t add value to my way of working.” (ID15); “For those with experience, you won’t really get much new out of it...I’d like the tips to be a bit more concrete.” (ID2). However, other professionals did report added value: “It gave me a much broader view of what I could pay attention to in a case...I think of many things, and at the same time the tool gives me even more things to consider.” (ID3), “Taking another perspective, another way of looking at the situation, gives you more tools to work with.” (ID8). At the same time, several participants mentioned greater potential for professionals with less experience: “For people with less experience, I think it could help, because it speeds up a process.” (ID7); “I think a lot of psychologists are still searching, so if you are starting to work with this population, it is definitely valuable that this exists.” (ID10); “I can imagine some former colleagues having a real ‘wow experience,’ for example, around grief, because they do not always think about that.” (ID17). This suggests that the perceived value of the tool may depend on users’ existing expertise and on what they expect the tool to provide. Less experienced professionals may benefit primarily from its educational and reflective structure, whereas more experienced users may require more efficient pathways and more case-specific decision support.

#### Category 5: Suggestions for Improvement

Priorities for further improvement focused primarily on shorter run-throughs, more concise advice at the end, and more concrete referral guidance: “It was very long; it could be shorter...or perhaps include an executive summary.” (ID8), “In the advice, be clearer about where, when, and to whom to refer.” (ID1). Participants also suggested more flexible response options: “There was little flexibility in the response options...it was too black-and-white.” (ID16), “Sometimes I felt that when selecting an answer, I would rather work with a gradation scale.” (ID18).

## Discussion

### Principal Findings

The present study had three aims: to identify the needs and questions of professionals working with people with a refugee background, to map these needs onto an initial conceptualization of IMPACCT as a digital decision-support tool, and to collect initial feedback on its relevance and usability. The analysis of 905 help desk questions showed that professionals sought support for a broad range of concerns. Trauma- and adversity-related concerns were prominent, followed by indicators of anxiety or depression and issues associated with acculturation. The semistructured interviews suggested that IMPACCT was generally perceived as relevant and useful, particularly for structuring reflection in complex cases and clarifying possible next steps. At the same time, the time investment required to use the tool was identified as an important barrier to its implementation.

The prominence of trauma- and adversity-related concerns is consistent with the elevated exposure of refugee children and families to potentially traumatic experiences before, during, and after migration. However, this pattern also requires careful interpretation. Grief was coded much less frequently than trauma, even though migration often involves bereavement and broader forms of loss, including family separation, loss of status, language, and routines [20]. This does not necessarily mean that professionals fail to recognize grief or loss in practice. Rather, grief-related processes may have been less visible in the help desk questions because professionals used trauma as a broad umbrella term or because the help desk format encouraged them to foreground the most urgent difficulties to obtain advice. The finding therefore points less to a confirmed blind spot than to the need for structured support that helps professionals differentiate between trauma, grief, adversity, and loss when formulating children’s needs.

A similar issue emerged for acculturation and cultural responsiveness. Although acculturation-related questions were present, they were often framed as individual difficulties with integration rather than as potentially stressful psychological and relational processes shaped by broader social conditions [21]. Likewise, cultural responsiveness was also not raised explicitly, even though it is widely recognized as a condition for equitable mental health care [22]. In addition, family- and school-related questions were present with moderate frequency, but professionals did not routinely refer to parental distress or trauma as possible drivers of child difficulties. These findings should again not be taken to mean that professionals were unaware of these dimensions. Rather, they suggest that such dimensions were less often foregrounded in help desk questions, which were by nature problem-oriented and focused on obtaining advice. This supports the need for a tool that prompts professionals to consider not only adversity and symptoms, but also acculturation, cultural meaning-making, family and school context, resilience, and protective factors.

IMPACCT was therefore not designed to simply reproduce the concerns most frequently raised in the help desk questions but to translate ecological and culturally responsive formulation into a practical, guided workflow. By prompting professionals to consider grief, acculturation dynamics, cultural interpretation, resilience, family and school context, and care pathways, the tool aims to support a broader formulation of children’s needs. Such attention to resilience and protective factors is important for gaining a comprehensive understanding and for facilitating the processing of trauma and loss [23,24].

The semistructured interviews revealed that most participants considered the tool’s content relevant, particularly the reflective questions and the guidance it provided when they were uncertain about next steps in a case. The main barrier identified was the required time investment. Participants also highlighted the need for shorter versions, concise summaries of advice, and clearer referral options. While highly experienced professionals reported gaining fewer new insights, others described the tool as a helpful guide in complex cases and a valuable learning resource. For broader implementation, some participants suggested that IMPACCT could be positioned as a training and supervision aid in first-line services and as decision support in ambulatory and inpatient care.

### Comparison With Prior Work

Our study builds on and extends earlier work on the assessment of mental health needs among refugee populations, which has already demonstrated the importance of moving beyond an exclusively psychiatric framework toward a more ecological and culturally sensitive approach to care [10]. IMPACCT operationalizes this perspective in a practical and accessible format by combining decision support with educational and reflective elements. To our knowledge, there is currently no comparable digital tool aimed specifically at strengthening cultural competence and systemic awareness among professionals working with refugee children. Existing screening tools, such as the Screening Tool for Asylum-seeker and Refugee Mental Health (STAR-MH) [25] or the Self-Report Questionnaire-5 [26], are primarily designed as diagnostic instruments, not as digital decision support tools. While valuable for detecting disorders such as depression or posttraumatic stress disorder, these tools do not have an educational function, are not interactive, and do not support professionals in broader case conceptualization.

### Limitations

The main limitation concerning the development of the decision support tool is its reliance on help desk questions. While this ensured a strong link with practice, the questions were not exclusively focused on children with a refugee background. In addition, interrater reliability for the help desk coding varied across categories and was lowest for *culture and migration*. This may reflect the conceptual complexity of this category and the fact that real-world help desk questions often combine cultural, migration-related, clinical, and systemic concerns. As the remaining help desk questions were single-coded after calibration, frequencies should be interpreted as indicative patterns rather than precise prevalence estimates, particularly for categories with lower agreement. Although the broader PACCT framework offered a counterweight, the possibility of remaining blind spots cannot be ruled out. A further limitation is that refugees were not directly involved in the development of the tool, meaning that their perspectives were included only indirectly. Finally, the first evaluation of IMPACCT relied on self-reported feedback from professionals and is therefore vulnerable to memory and social desirability bias.

### Future Directions

Participant feedback points to several priorities for further development. First, IMPACCT should be made less time-consuming, for example, through shorter versions, concise summaries of advice, and clearer referral options. Second, future versions should be evaluated not only for usability and acceptability but also for their impact on professional reflection, cultural competence, case formulation, and referral decision-making. Third, the perspectives of children, families, and people with a refugee background should be incorporated more directly, as the current development process relied primarily on professionals’ questions and feedback.

Future research could also explore whether more conversational forms of digital support would make the tool easier to use in practice. One promising avenue is the development of an IMPACCT chatbot powered by AI, which would enable professionals to discuss cases in a more natural, conversational manner while receiving quicker and more tailored feedback.

### Conclusions

This study suggests that professionals working with children and young people with a refugee background need support not only in recognizing trauma- and adversity-related concerns but also in formulating these concerns within broader contexts of grief, acculturation, cultural meaning-making, family and school systems, resilience, and care pathways. IMPACCT represents an initial attempt to translate this broader ecological and culturally responsive perspective into a practical digital decision-support tool. Its potential value lies not in replacing professional judgment but in helping professionals structure reflection, recognize less foregrounded dimensions of a case, and clarify possible next steps.

At the same time, the findings underline that such digital decision support must remain both clinically useful and practically feasible. Further development should therefore focus on making IMPACCT more concise, strengthening referral guidance, incorporating the perspectives of children and families with a refugee background, and evaluating whether the tool improves professional reflection, cultural competence, and decision-making in practice. More broadly, this study highlights the potential of digital decision support to help professionals make more contextualized and better-informed decisions in refugee mental health care.

## Supplementary material

10.2196/86809 Multimedia Appendix 1 PACCT background.

10.2196/86809 Multimedia Appendix 2 IMPACCT background.

10.2196/86809 Multimedia Appendix 3 Interview guideline.

10.2196/86809 Checklist 1 SRQR checklist.

## Abbreviations

*DSM-5*: *Diagnostic and Statistical Manual of Mental Disorders, 5th Edition*
IMPACCT: Implementing PACCT through a Culturally Contextualized Tool
PACCT: Psychiatry Assisting the Culturally Diverse Community in Creating Healing Ties approach
SRQR: Standards for Reporting Qualitative Research
STAR-MH: Screening Tool for Asylum-seeker and Refugee Mental Health
