# Supplementary material for: A Digital Tool for Culturally Responsive Reflection and Case Conceptualization in First-Line Services to Support Children With a Refugee Background: Development and Usability Study
Source: JMIR Form Res. 2026 Oct 15;10:e86809. doi: 10.2196/86809 (PMC13671483; doi:10.2196/86809)
Supplement: Supplementary file 1 [file formative-v10-e86809-s001.pdf]

Appendix 1 – PACCT background

Psychiatry Assisting the Culturally Diverse Community in Creating Healing Ties approach (PACCT) was created as a community-based intervention model to work with culturally diverse families PACCT emphasizes that effective transcultural, culturally sensitive and trauma-sensitive care always starts from and engages with the child's community. It is within this community that the values, beliefs and frames of reference that shape a child's life are formed. The closer one remains to these values and beliefs in defining the problem and in seeking solutions, the more effective care is. Furthermore, trauma and trauma treatment needs structure and containment. Moreover, when defining the problem and seeking for solutions, the focus is on what it still working and enhancing the resilience children are witnessing of. PACCT rests on three thematic pillars. The *first pillar* is outreach. Children with a refugee or migration background tend to have less access to mental healthcare compared to their peers, which limits their developmental chances. It is therefore important to bring care closer to these children and to approach them through their own frames of reference rather than relying solely on a Western perspective. The *second pillar* emphasizes the importance of intercultural dialog. The acculturation process that children undergo can be both enriching and challenging. To help them find balance between multiple cultural contexts, caregivers should engage in conversation about the child's and family's frames of reference and belief systems. The *third pillar* highlights the creation of healing connections and a focus on resilience. Migration often means leaving behind established social networks, which can lead to isolation. PACCT therefore recognizes that a child's well-being is closely tied to social participation, whether through school, work, hobbies, or community life. It builds on the understanding that children possess significant resilience and therefore seeks to amplify what remains intact rather than focusing solely on what is damaged, aligning with principles of positive psychology.
