# Supplementary material for: A Digital Tool for Culturally Responsive Reflection and Case Conceptualization in First-Line Services to Support Children With a Refugee Background: Development and Usability Study
Source: JMIR Form Res. 2026 Oct 15;10:e86809. doi: 10.2196/86809 (PMC13671483; doi:10.2196/86809)
Supplement: Supplementary file 2 [file formative-v10-e86809-s002.pdf]

Appendix 2 - IMPACCT background

IMPACCT is a responsive website, through which participants first submit basic background information, including their professional role, care zone, the child's age, and the child's country of origin. Before proceeding to the case exploration, users are guided through a brief cultural sensitivity reflection. This section asks whether the professional has reflected on their own cultural position, whether the family understands the Western care context, and the possible need for an interpreter. Each question includes an explanatory *"Why is this important?"* pop-up and the tool offers additional resources on cultural sensitivity and interpretation where relevant.

The next phase of the tool is structured around an ecological perspective following Bronfenbrenner [1]. Rather than focusing exclusively on the child, IMPACCT invites professionals to map concerns, stressors, and resources across the child's broader context. This is important because refugee children's wellbeing may not only be shaped by individual vulnerabilities or pre-migration trauma, but also by post-migration stressors, including acculturation processes, legal uncertainty, access to supportive networks, basic needs, and functioning across social, family, and school domains. Throughout this section, explanatory pop-ups again clarify the ecological layer addressed at each step and explain the rationale for the questions posed (Figure 1).

This ecological mapping is organized across several layers. At the individual level, IMPACCT explores the child's age, migration history, presenting concerns and the timing of difficulties. The microsystem focuses on the child's immediate environment, including social, family, and school functioning, basic needs, parental presence, parental difficulties, and informal networks. The mesosystem addresses connections between these immediate contexts, such as whether concerns are shared by parents, professionals or community members, and whether formal and informal networks collaborate. The exosystem includes contextual factors that indirectly affect the child, such as residence status, and safety in the neighborhood, and stress related to the events in the country of origin. Finally, the macrosystem examines broader social influences, such as racism, prejudice, and cultural tensions.

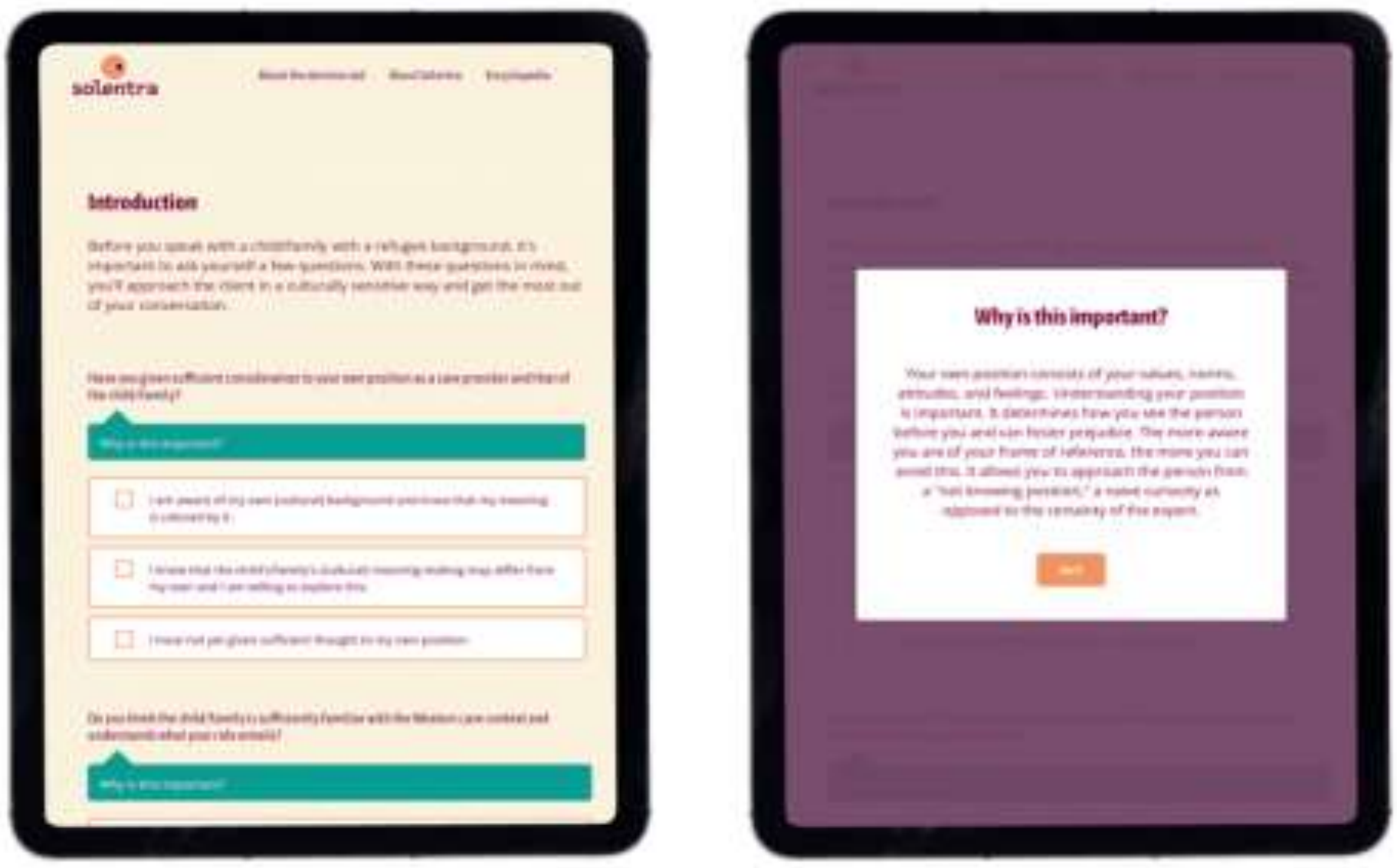

Figure 1. Multiple choice questions including the option to have key concepts clarified through pop-ups. The language in the screenshots has been translated from Dutch to English for publication purposes.

After this ecological mapping, the tool explores four central themes: Acculturation, Grief, Trauma, and Resilience (Figure 2). These sections prompt professionals to consider how the child and family are coping with cultural transition, loss and separation, potentially traumatic experiences before, during, or after migration, and available protective factors. The tool also encourages attention to parental support, family trauma, signs of complicated grief, coping strategies, trusted adults, social support, and community participation.

The tool concludes with personalized feedback (Figure 2), helping professionals distinguish expected migration-related stress from concerns requiring additional support and offers practical suggestions related to adjustment, safety, basic needs, parental involvement, resilience, and referral. This report can subsequently be printed and exported as a PDF. IMPACCT also includes an encyclopaedia with background information on themes such as acculturation, grief, trauma, resilience and stress reduction.

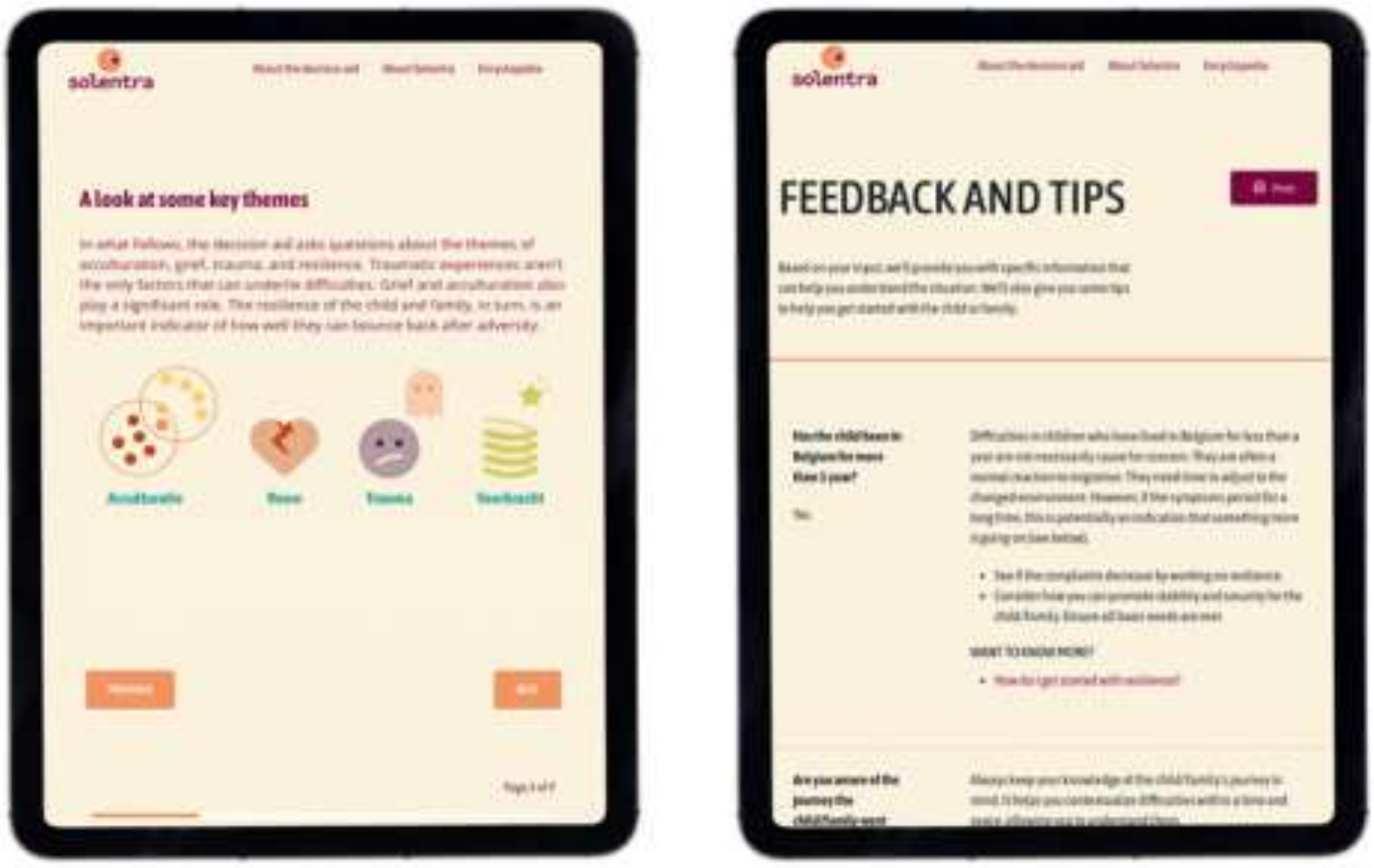

Figure 2. Introduction of key themes and final page of IMPACCT, summarizing answers and offering concrete feedback and tips. The language in the screenshots has been translated from Dutch to English for publication purposes.

References

1. Bronfenbrenner U. Toward an experimental ecology of human development. Am Psychol. 1977;32:513–531.
