# Supplementary material for: A Digital Tool for Culturally Responsive Reflection and Case Conceptualization in First-Line Services to Support Children With a Refugee Background: Development and Usability Study
Source: JMIR Form Res. 2026 Oct 15;10:e86809. doi: 10.2196/86809 (PMC13671483; doi:10.2196/86809)
Supplement: Supplementary file 3 [file formative-v10-e86809-s003.pdf]

Appendix 3 – interview guide

Socio-demographic information

1. What is your current profession and/or position?
2. What gender do you identify with?
3. What is your age?
4. How many years of professional experience do you have?
5. To what extent do you have previous experience with trauma in children with a refugee background?

User Experience

1. How did you experience using the tool?

2. Do you find the tool user-friendly?
   -> If so, why?
   -> If not, why not?

3. What did you find most useful about the tool?

4. What aspects of the tool would you like to change/improve based on your first experience?

Content and impact

1. Were all the questions clear?
   a. If not, which ones?

2. Were there any questions you couldn't answer?
   a. If so, which ones?

3. Does the advice help you to get started?
   a. If so, why?
   b. If not, why not?

4. Do you feel more competent to deal with this target group?

5. Do you look differently at the problems presented after going through the tool?

6. Would you possibly make a different decision to act after using the tool (e.g. do not refer to a specialized psychologist but take it yourself, a solution other than exploring therapy...)?

Practical relevance

1. Would you go through the decision tool for every case?

    a. Why or why not?

2. How often would you go through the tool?

3. Do you see the tool as an added value in the wider field?
    a. If so, why?
    b. If not, why not

4. Would you recommend the tool to your own colleagues?
    c. If so, why?
    d. If not, why not?

5. Would you or your organization be willing to pay for access to the tool?
    e. If so, why?
    f. If not, why not?

Conclusion

1. Do you have any final thoughts that you would like to share?
